# The relationship between subjective difficulty in interoceptive processing and accuracy of heartbeat perception in autistic individuals

**DOI:** 10.1007/s44192-024-00065-6

**Published:** 2024-04-18

**Authors:** Chihiro Itoi, Yuta Ujiie, Yuuki Ooishi, Makio Kashino

**Affiliations:** 1 NTT Communication Science Laboratories, 3-1 Morinosato Wakamiya, Atsugi, Kanagawa 243-0198 Japan; 2 College of Contemporary Psychology, Rikkyo University, 1-2-26 Kitano, Niiza-shi, Saitama, 352-8558 Japan; 3 Division of Psychology, Institute of Human Sciences, University of Tsukuba, 1-1-1 Tennodai, Tsukuba, 305-8577 Japan; 4 Research Organization of Open Innovation and Collaboration, Ritsumeikan University, 2-150 Iwakura-cho, Ibaraki, 567-8570 Japan

**Keywords:** Autism, Interoception, Interoceptive sensory questionnaire (ISQ), Heartbeat counting task (HCT)

## Abstract

Most autistic people experience difficulties in sensory processing, including interoceptive processing. For example, they often report subjective difficulties in the interoceptive processing of interoceptive input, such as difficulty in interpreting bodily signals, including hunger, thirst, and fatigue. However, whether these subjective interoceptive difficulties are from underlying problems in interoceptive accuracy remains unclear. This study investigated the relationship between subjective interoceptive difficulty and behavioral interoceptive accuracy in autistic adults and a control group. Subjective interoceptive accuracy was measured using an interoceptive sensitivity questionnaire, and behavioral interoceptive accuracy was measured using a heartbeat counting task. The results showed no significant relationship between subjective interoceptive difficulty and behavioral interoceptive accuracy in the autistic or control groups. This suggests that subjective interoceptive difficulty and behavioral interoceptive accuracy reflect different aspects of interoceptive processing. One possible interpretation is that autistic adults can identify individual local sensory inputs, such as heartbeats, however, they have difficulty integrating multiple inputs and recognizing internal body states such as hunger and fatigue**.**

**Supplementary Information:**

The online version contains supplementary material available at 10.1007/s44192-024-00065-6.

## Introduction

Approximately 90% of the individuals with autism spectrum disorder (ASD) have experienced atypical sensory processing [1, 2]. Studies on atypical sensory processing in autistic individuals have primarily focus on exteroceptive sensory processing characteristics, which refers to the processing of sensory stimuli from outside the body, such as vision and audition [3, 4]. However, recently, the differences in subjective difficulty in interoceptive processing of interoceptive input in autistic individuals have been gaining attention [5, 6]. Interoception refers to the perception of internal bodily information, such as the state of one's internal organs and body fluids, and contributes to allostasis by providing information on visceral processes such as hunger, pain, temperature, and thirst. Studies based on autobiographies [7] and interviews [8] on interoceptive information processing in ASD suggest that autistic individuals are less responsive to internal body information such as reduced awareness of thirst. Additionally, clinical implications include a high rate of comorbidities of physical disabilities such as gastrointestinal systems [8] and migraine headaches [9]. Hence, it is crucial to intervene appropriately in the atypical processing of interoceptive information to maintain the physical health of autistic people.

Furthermore, interoception is widely associated with physical information and cognitive, emotional, and affective functions. Social and emotional regulation problems observed in autistic individuals may be related to challenges interpretating their internal bodily signals [5]. For example, difficulty with interoceptive perception is associated with alexithymia (difficulty identifying and expressing emotions), which commonly coexists with autism [10].

There are various methods for measuring interoceptive processing ability [11, 12]. Murphy et al. (2019) proposed classifying individual differences in the perception of the body’s internal state based on how interoception is measured (using an objective measure or subjective self-reporting) and what aspect of interoception is measured (such as accuracy of interoceptive perception or attention to interoceptive signals). In other words, interoceptive information processing is measured in different ways: experimentally measured interoceptive accuracy is treated as an objective behavioral aspect, and one’s usual perceptual experience is treated as a subjective aspect measurable by questionnaires. Studies of interoceptive processing in autistic individuals have used both behavioral and self-report measures.

Most objective interoceptive research on autism focuses on cardiac interoception. Historically, two different tasks have been used for assessing the interoceptive accuracy of heartbeat sensations: the heartbeat counting task (HCT) [13] and the heartbeat discrimination task (HDT) [14]. The HCT requires that participants to count their heartbeats within a specific period, and a comparison between the reported and actual measured number of heartbeats is performed to assess interoceptive accuracy. The HDT requires participants to judge whether their heartbeat sensations are simultaneous with exteroceptive stimuli (e.g., tone) presented at different delays. Previous studies on the accuracy of heartbeat perception in autistic individuals, shown that they have reduced interoceptive accuracy on HCT tasks, but no significant differences in HDT tasks than the control group [15–17]. A study in non-ASD individuals also found no association between HCT and HDT results, suggesting that each task measures different aspects of interoceptive accuracy; HCT is influenced by prior heartbeat knowledge, while HDT requires combined information of both external and internal sensory inputs [18].

In studies on subjective interoceptive sensitivity in ASD, the typically used self-report measure is the Body Perception Questionnaire (BPQ) [19]. The BPQ measures beliefs about one’s degree of attention to interoceptive signals [12]. The BPQ comprises items for awareness of localized bodily states associated with anxiety (items such as “during most situations, I am aware of how hard my heart is beating”), and significantly high scores are perceived as maladaptive states [20]. Studies also report significantly higher BPQ scores for autistic adults than in the control group [15, 21]. This finding suggests that subjective attention to interoceptive information is high in autistic adults for hypersensitivity. However, as mentioned earlier, autistic individuals also exhibit hyposensitivity, which is difficulty identifying and describing their own interoceptive information. The Interoception Sensory Questionnaire (ISQ) assesses the usual experiences of interoceptive processing in autistic adolescents and adults [22]. This questionnaire comprises items that refer to ASD-specific difficulties and confusion in interoception, which were captured in semi-structured interviews with autistic adults regarding their internal sensory processing. For example, the items include the following sentences: “if interoceptive bodily signals are not extremely hypo- and/or hyper-, those signals are difficult in identifying and describing”, “reliance on external cues”, or “reduced emotional and motivational components to work on physical states”. Studies consistently show that autistic adults have higher ISQ scores; that is, they experience more subjective interoceptive difficulties and confusion than individuals in control group [21, 22]. In a study with ASD and control groups, the ISQ was significantly positively correlated with the BPQ, suggesting that hyposensitivity and hypersensitivity to interoceptive inputs may exist simultaneously in the same person [21].

In the relationship between subjective and objective interoception, autistic individuals exhibit a disparity between the accuracy of their objective interoception and the sensibility of their subjective interoception, which has been linked to anxiety [15, 23]. Specifically, a study on autistic adults showed lower accuracy on the HCT and heightened interoceptive sensibility on the BPQ than the control group [15]. Nevertheless, the BPQ scores of autistic children were not significantly different from those of the control group [23]. Despite this, the BPQ scores were consistently positively correlated with anxiety, suggesting that the BPQ measures maladaptive forms of anxiety-related interoceptive attention traits.

However, studies do not show a direct relationship between the subjective difficulty in identifying and describing interoceptive information and lower interoceptive accuracy. Therefore, this study specifically focuses on exploring the relationship between the subjective interoceptive confusion and behavioral accuracy of interoceptive information processing in ASD. We examined the relationship between the HCT, which is lower in autistic adults [15], and the ISQ, a subjective measure of difficulty in identifying interoceptive information. Our hypothesis posits an inverse correlation between subjective interoceptive difficulty and behavioral accuracy in autistic adults. Precisely, we predict that autistic participants who report significant subjective confusion in interoceptive experiences demonstrate lower behavioral accuracy in HCT. To test this hypothesis, we investigated the relationship between subjective and behavioral interoceptive accuracy in autistic adults.

## Methods

### Participants

Twenty autistic adults (14 females; ASD group) and twenty in the control group (12 females; control group) participated in this study. All autism participants had received a formal diagnosis (DSM-IV-TR or DSM-5) from a psychiatrist. Participants in the ASD and Control groups were matched for demographics with no significant group differences in age (ASD group: Mean age = 30.8 ± 7.5, Control group: Mean age = 27.8 ± 8.1), and IQ (ASD group: Mean FIQ = 104.4 ± 15.4, Control group: Mean FIQ = 110.9 ± 14.7) (assessed by WAIS-III or IV).

Participants in the control group had no history of psychiatric illness or neurological disorder. Fourteen of the twenty participants in the ASD group were using one or more of the following medications: anti-depressants (four participants), hypnotic drugs (two participants), anti-anxiety drugs (six participants), antipsychotic drugs (four participants), anti-epileptic drugs (two participants), and central nervous system stimulants (eight participants). Table 1 presents detailed information on the participants.

**Table 1:** Participants demographic information

	ASD group	Control group	p-value*
Sex (male/female)	6/14	8/12	0.74
Age (years)	30.8 ± 7.5	27.8 ± 8.1	0.23
Full-Scale IQ^†^	104.4 ± 15.4	110.9 ± 14.7	0.17
Verbal comprehension	110.3 ± 16.0	109.9 ± 13.0	0.94
Perceptual-Reasoning (Perceptual Organization)	100.6 ± 15.1	104.2 ± 14.8	0.45
Working Memory	102.5 ± 27.7	111.1 ± 14.7	0.23
Processing Speed	95.0 ± 18.8	110.0 ± 12.6	< 0.01
Medications (n / %)			
Anti-depressants	4 (20%)	N.A	
Anti-psychotics	4 (20%)	N.A	
Anti-anxiety	6 (30%)	N.A	
Anti-epileptic drugs	2 (10%)	N.A	
Hypnotic drugs	2 (10%)	N.A	
Central-nervous-system stimulants	8 (40%)	N.A	

*Difference in sex is tested using a chi-squared test, and the differences in other contents are tested by a t-test

^†^The participants for the control group were all assessed by WAIS-IV, and the participants for the ASD group were assessed by WAIS-IV or WAIS-III

### Subjective measurements

The ISQ [22] measured the hyposensitivity of interoceptive information processing. It comprises 20 items that assess confusion regarding bodily signals (such as difficulty in understanding hunger or thirst) using a seven-point scale ranging from 'strongly disagree' to 'strongly agree.’ The total possible ISQ score ranged from 20 to 140.

The severity of autism spectrum traits was measured using the Japanese version of the Autism Spectrum Quotient (AQ) [24], which assesses autistic traits across five domains: social skills, communication, attention to detail, attention switching, and imagination. It contains 50 items rated on a four-point Likert scale, ranging from “definitely disagree” to “definitely agree.” The total possible AQ scores range from 0 to 50. Table 2 presents the results of the questionnaire.

**Table 2:** Results of the questionnaires

Variables	ASD group	Control group	t-test
ISQ	80.9 (25.8)	42.8 (19.5)	p < 0.0001
AQ Total	33.2 (7.6)	18.1 (6.5)	p < 0.001
Social skill	6.60 (2.9)	3.35 (2.0)	p <0.001
Attention switching	7.45 (1.8)	4.40 (2.2)	p < 0.001
Attention to detail	6.30 (1.8)	4.70 (2.3)	p =0.02
Communication	7.15 (2.4)	2.65 (2.3)	p <0.001
aImagination	5.70 (2.0)	3.00 (1.6)	p < 0.001

### Behavior measurement

We used the HCT [13], which measures behavioral interoceptive accuracy. Participants were seated and their heartbeats were counted during three randomized time windows of different durations (25, 35, and 45 s) and then reported the number of heartbeats they detected to the experimenter at the end of each trial. Actual heartbeats were recorded using a physiological data monitoring system (BIOPAC MP150, BIOPAC Systems, United States). Before starting the HCT, participants were given the following verbal instructions: “Count the number of heartbeats you feel without touching your body from when you hear 'start' to when you hear 'stop'. Count in your head, and I will ask how many heartbeats you felt afterward”. To determine interoceptive accuracy (IA) scores, heartbeat-counting scores were calculated on a trial-by-trial basis using the ratio of perceived to actual heartbeats$1-|{\text{nbeatsreal}}-{\text{nbeatsreported}}|/(({\text{nbeatsreal}}+{\text{nbeatsreported}})/2)$ [11]: and then averaged to produce a mean heartbeat-counting score.

### Physiological measurement

Electrocardiograms (ECGs) were used to measure R waves during HCT experiments. The analog data were amplified and digitized using a BIOPAC MP150 (BIOPAC Systems, United States). The sampling rate was 1,000 Hz. As in a previous study [25], R-wave detection was performed using AcqKnowledge analysis software (BIOPAC MP150, United States). The data were visually screened to eliminate any inappropriate R-wave detection, which may be from artifacts, such as movement.

To adjust the measurement timing of R waves by BIOPAC MP 150 with the HCT, we pushed the start and the stop buttons in Acqknowledge analysis software following to our call ‘start’ and ‘stop’ noted above in the Behavior section.

### Data analysis

The number of counted or actual heartbeats within the experimental time window (25, 35, or 45 s) was reduced to a value within 60 s. For example, the x beats within 45 s were reduced to x * 60/45 [beats/minute]. Normalized heartbeats were used for the statistical analyses. The differences between the ASD and control groups in the IA score, actual heartbeats (beats/minute), ISQ score, and AQ score were determined using independent t-tests. Path analyses of interoceptive accuracy, actual heartbeat, ISQ score, and group (ASD or Control) were performed using multivariate regression and partial correlation analyses.

All statistical analyses were conducted using the R software (version 3.6.1., for Windows; RStudio Team, PBC, Boston, MA).

## Results

### Group differences in behavioral interoceptive accuracy, and actual heartbeats

**Fig. 1:**
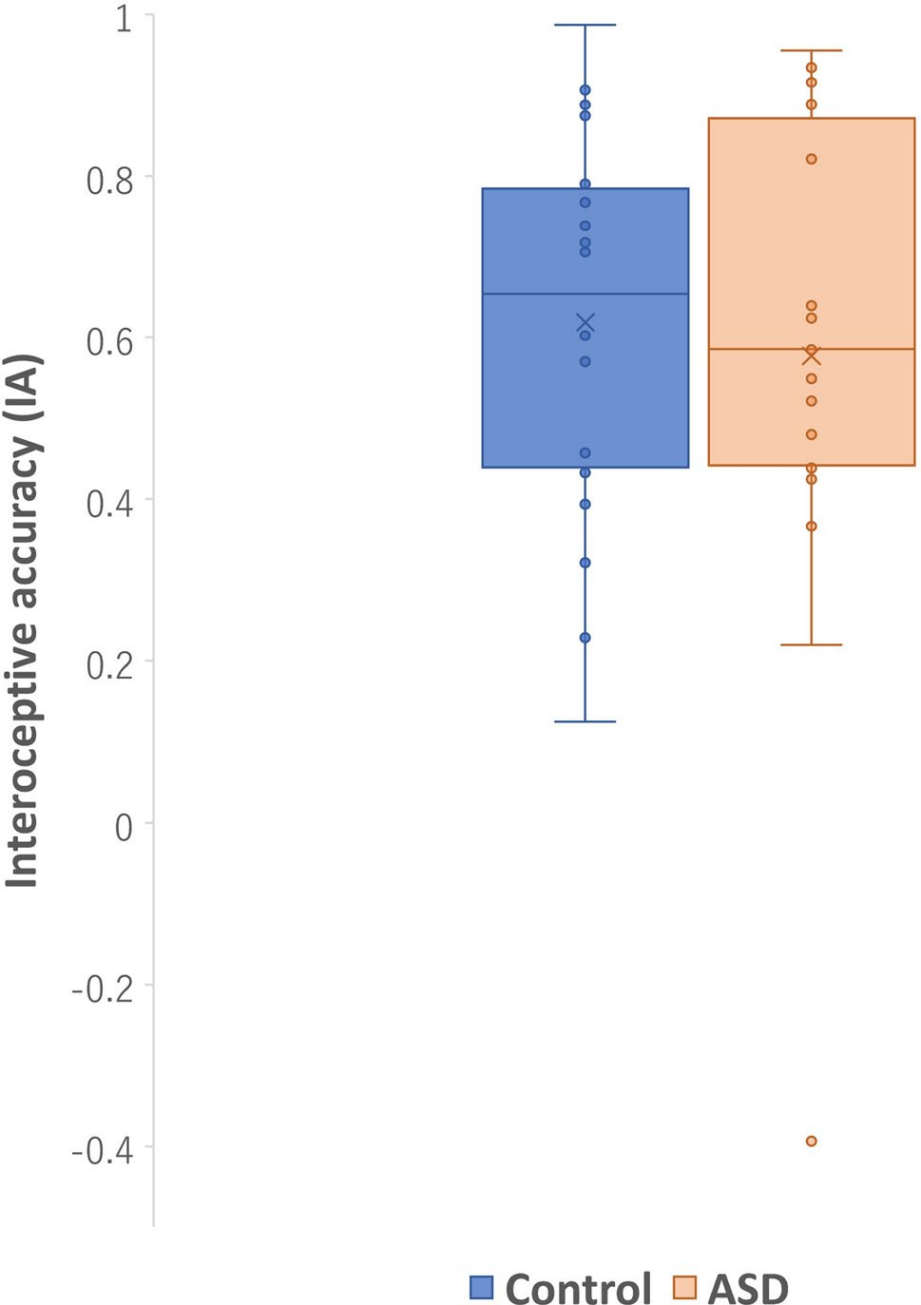
Group differences in IA score between ASD and control groups

**Fig. 2:**
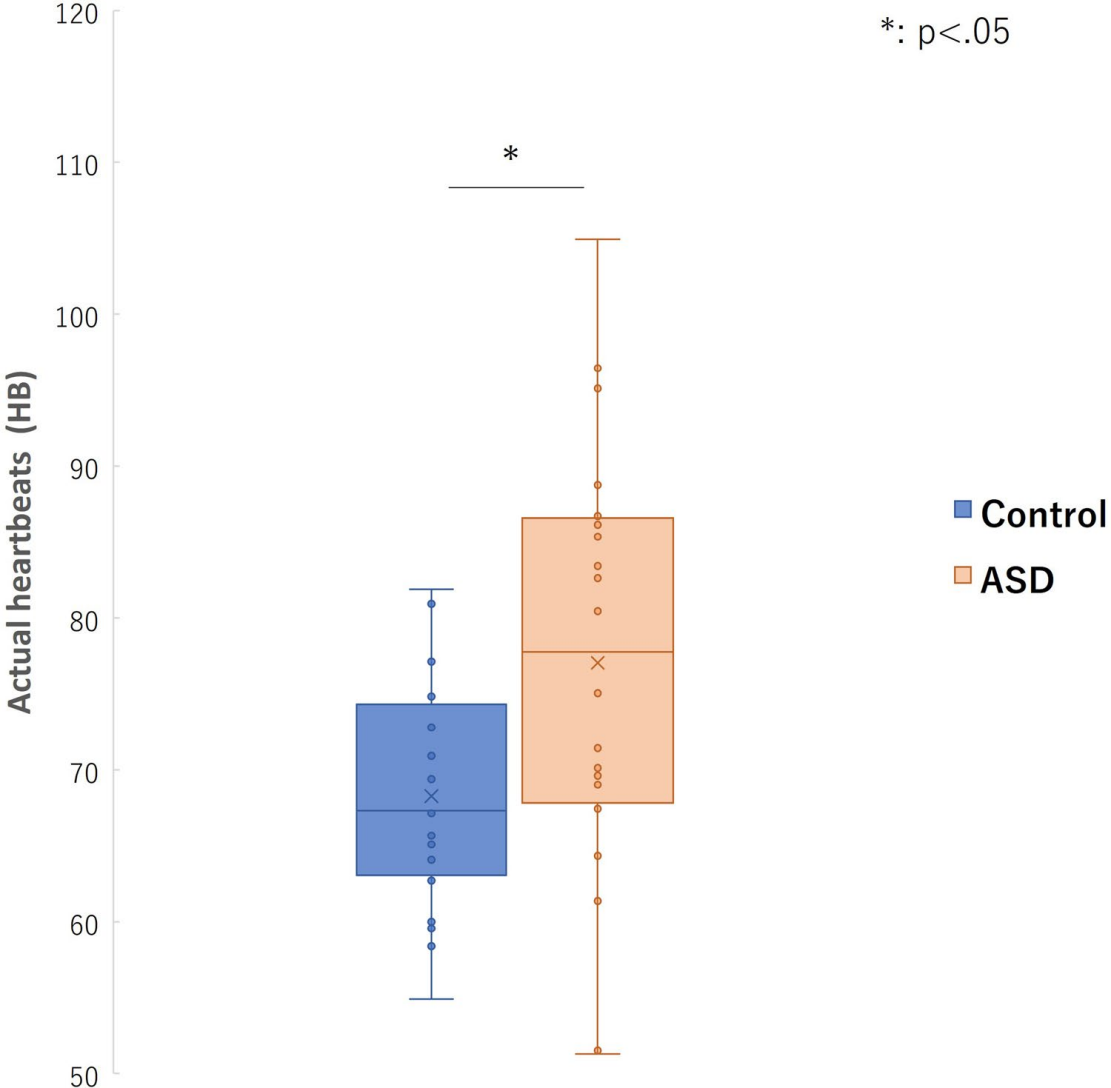
Group difference in actual heartbeats between ASD and control groups

Regarding HCT, there were no significant differences in IA scores between the ASD and Control groups (t (38) = 0.47, p = 0.63) (Fig. 1). In addition to HCT, the actual heartbeat (HB) was statistically evaluated. The ASD group had significantly more heartbeats (t (38) = 2.41, p = 0.02, Cohen’s d = 8.35) than the control group (Fig. 2). There were no significant differences in the IA scores (p = 0.30) and actual heartbeats (p = 0.65) between individuals who received medication (14 participants) and those who did not (6 participants) in the ASD group.

### Relationship between subjective, behavioral interoceptive accuracy and actual heartbeats

To perform a path analysis of the ISQ score, IA score, actual HB, and group (ASD or Control), a combination of a multivariate regression analysis and partial correlation analysis was performed.

Multivariate regression analysis revealed significant effects of group variables on dependent variables. For the ISQ score, the group variable had a significant positive effect (β = 1.28, SE = 0.24, t(38) = 5.27, p < 0.001). Similarly, the group variable showed a significant positive effect on HB (β = 0.72, SE = 0.30, t(38) = 2.41, p = 0.02). However, it did not significantly affect the IA score (β = − 0.15, SE = 0.32, t(38) = 0.48, p = 0.64). These results indicate that the group variable significantly impacted the actual heartbeats and ISQ scores, but it did not significantly affect the IA score.

**Fig. 3:**
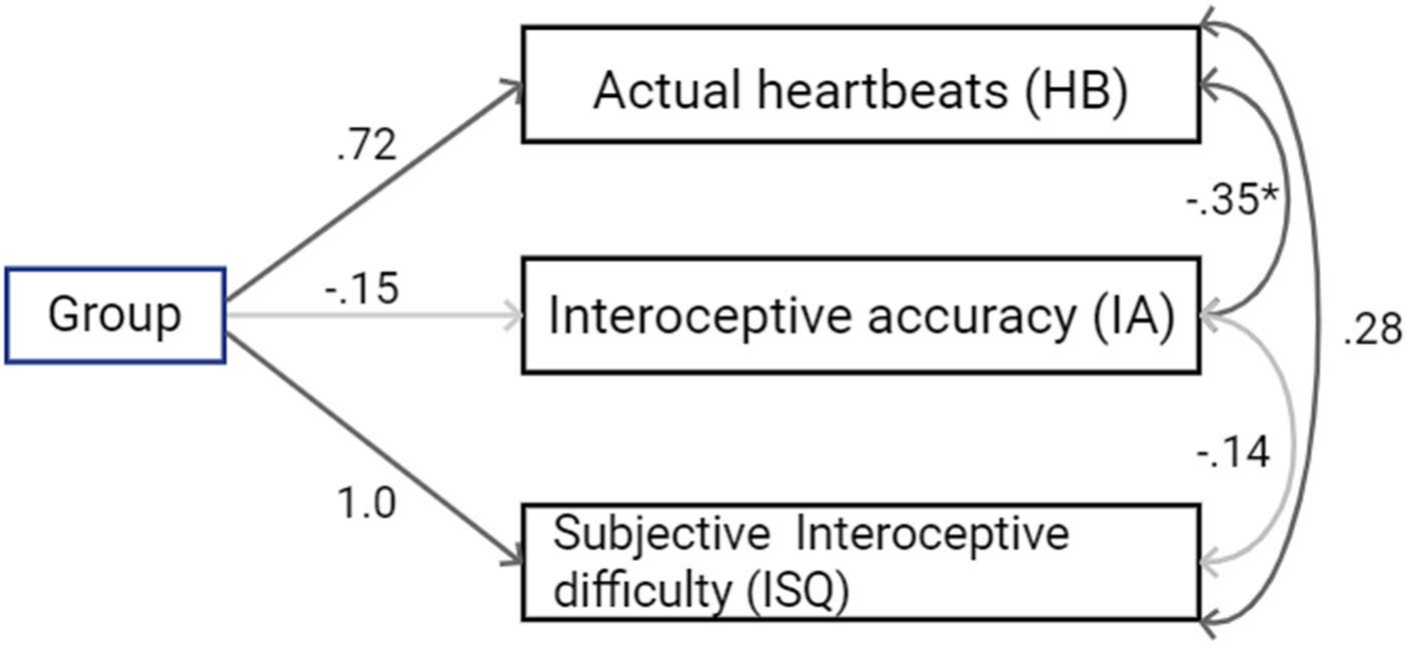
A path diagram illustrates the relationships between the presence of diagnosis and actual heartbeats (HB), interoceptive accuracy (IA), and ISQ score determined by using multivariate multiple regression and correlation analysis

Subsequently, a partial correlation analysis revealed a significant negative correlation between actual heartbeats and the IA score (r = − 0.35, p = 0.03) after controlling for the group variable. However, no significant correlation was observed between ISQ score and HB (r = 0.28, p = 0.08) or between ISQ score and IA score (r = − 0.14, p = 0.41). The overall path diagram is shown in Fig. 3.

## Discussion

This study examined the relationship between subjective interoceptive difficulty and interoceptive behavioral accuracy in autistic adults and a control group. The results showed no significant relationship between subjective interoceptive difficulty and behavioral interoceptive accuracy in the ASD or the control groups. These results contradicted our hypothesis, which predicted a significant correlation between ISQ and IA scores. There are several possible explanations for this.

One possibility is that subjective interoceptive difficulty and behavioral interoceptive accuracy reflect different aspects of interoceptive processing. The ISQ measures difficulty in perceiving and dealing with overall bodily conditions, such as fatigue and hunger [22]. In contrast, HCT, measures the accuracy of heartbeat perception, which is a local aspect of interoceptive information. Therefore, these two measures may not be substantially correlated. Studies on subjective interoceptive processing in autistic people show how much they are aware of internal bodily signals [15, 17, 23]. Using interoceptive awareness questionnaires (e.g., body perception questionnaire; BPQ [19], a study showed that autistic adults notice localized bodily symptoms such as fast heartbeat, dry mouth, and sweating palms [15]. In contrast, the ISQ assesses difficulties specifically for autistic people in perceiving more integrated internal bodily signals, such as thirst and hunger (e.g., thirst refers to the integration of multiple physiological signals such as thirst in the mouth, increased viscosity of saliva, low blood, and increased osmotic pressure; [26], and in knowing how to deal with them [22]. These features (i.e., noticing localized bodily symptoms but having difficulty paying attention to whole integrated bodily states) may be associated with the weak central coherence (WCC) theory [27], which posits that the autistic people have a bias toward local information and have difficulty integrating and processing it. Although the WCC theory was initially developed to explain the characteristics of exteroceptive sensory processing, it can be applied to interoceptive sensory processing in autistic people [5, 21]. Evidence from neural circuits shows lower long-range and greater short-range connectivity of autistic individuals [28, 29]. Furthermore, the connectivity of anterior insular cortex, where interoceptive and exteroceptive information converge [30], shows atypical functional and structural connectivity in autistic individuals [31]. Therefore, they may notice local parts of internal signals (e.g., heartbeats), while having difficulty paying attention to all integrated bodily states. This may explain why the ISQ score was not related to the IA score on HCT, which measures a measure of local parts of the internal signals. In other words, the subjective difficulties experienced by autistic adults with interoceptive information may not be reflected in the accuracy of heartbeat perception. As described by Murphy et al. (2019), interoception is assessable on the four dimensions of a 2 × 2 factorial model: accuracy or attention, both objectively and self-reported. Regarding subjective measures of interoception, accuracy and attention measure different aspects. For example, the recently developed Interoception Attention Scale (IATS) measures different aspects of interoception compared to the Interoception Accuracy Scale (IAS), a subjective measure of accuracy [32]. Although there are multiple interoception subjective measures, selecting a scale appropriate for the study’s purpose is crucial, considering the aspects of attention or accuracy being measured. Hence, the question items of several commonly used subjective measures of interoception (e.g., BPQ, Multidimentional Assessment of Interoceptive Awareness; MAIA [33], Body Awareness Questionnaire: BPQ [19]) are also supported since they do not show consistency [34]. In the present study, the ISQ was chosen as a subjective measurement, focusing on the difficulty of noticing interoceptive information in daily situations for autistic adults. However, as a previous study showed that the ISQ significantly positively correlates with the BPQ, an aspect of interoceptive attention [21], the ISQ may measure the aspect of interoceptive attention and did not confirm a direct relationship with the HCT, which measures interoceptive accuracy. Future studies should examine the relationship between the ISQ, IATS and IAS to elucidate further which aspects of interoception the ISQ measures.

Moreover, this study showed no difference between the ASD and control groups in the relationship between objective (HCT) and subjective measures (ISQ) of interception. A study on an autistic group showed that anxiety was related to the difference between subjective and objective interoceptive measures [15, 23]. To examine the differences in the relationship between subjective and objective measures of interoception in the clinical and healthy groups, the relationship between the disparity of subjective and objective measures and associated symptoms must be examined. For example, a study revealed the relationship between subjective interoceptive measurement (MAIA) and objective task (HCT) in patients with schizophrenia, and the disparity between the accuracy of their objective interoception and their subjective interoception was linked to positive symptoms, especially delusions [35].

Another possibility is that the number of heartbeats reported by participants in the HCT reflects bottom-up interoceptive information (e.g. heartbeat detection) and includes top-down information, such as memories and knowledge about heartbeats [36]. The present study found a significant difference in the actual number of counted heartbeats between the ASD and control groups. This may be from trait-like biological and psychological factors, which may have affected the participants’ perceived and counted/ or reported number of heartbeats. While only a few studies using ASD groups have examined interoceptive accuracy, some found no group differences in the number of heartbeats [15], whereas others have indicated faster heartbeats in the ASD group [37]. However, many previous studies investigating autonomic responses have consistently reported faster heartbeats in autistic individuals [38–40]. Our results show that the ASD group exhibited faster heartbeats, which corroborates the findings of previous studies. In other words, it is possible that autistic people, as well as control individuals, may have knowledge and experience of their own heart rate. This may explain why there was no significant difference in the HCT. Regarding the accuracy of the number of heartbeats, studies with autistic children have shown lower accuracy [23, 41, 42], while previous studies with adults did not show any differences from control groups [41]. It could be assumed that autistic adults might perform this task in diverse ways by using their experience and knowledge of their bodies, however, further research is needed to test this hypothesis.

In summary, this study indicates that an ASD diagnosis is associated with subjective interoceptive difficulties, but not with behavioral interoceptive accuracy. This suggests that autistic individuals may experience interoceptive difficulties in various ways. For example, they may have difficulty perceiving or understanding their integrated bodily state, but may perceive specific local bodily signals without difficulty, such as heartbeats. These features may manifest as hypersensitivities or hyposensitivities in bodily sensations, depending on their mind and body states. Our findings have several implications for future research. First, it is critical to consider both global and local aspects of interoception when studying interoceptive processing in autistic individuals. Second, further research is needed in order to develop more comprehensive measures of the global aspects of interoception for autistic people with interoceptive difficulties.

## Limitations

The study has some limitations.

First, it is essential to consider that factors such as IQ have been associated with IA in HCT, both in typically developing individuals [43] and in autistic individuals [37]. Therefore, the absence of a difference in HCT in our study may be from a lack of significant group differences in IQ scores.

Second, although the ISQ was used in this study as a subjective interoceptive measure, it may have been appropriate to use the BPQ to measure interoceptive perception in the control group, given that the ISQ is a questionnaire designed based on symptoms in autistic adults. Furthermore, a significant association between AQ and IA was found in the control group, but no significant correlation was found in the ASD group (referred to as supplemental information). Future studies should examine the relationship between continuous autistic traits and subjective and objective interoceptive information processing in typically developing individuals.

Third, the sample in the present study was relatively imbalanced regarding sex, with more females than males. This is in contrast to the actual male-to-female ratio of ASD prevalence. This imbalance may have been caused by the recruitment through social media. However, the sex ratio in the control group matched that of the ASD group, which reduced the statistical significance of the sex imbalance. Additionally, the present study did not include enough participants to draw firm conclusions about the relationship between interoceptive difficulty and behavioral interoceptive accuracy in autistic females and males. Future studies should focus on recruiting a larger sample, including both males and females, to investigate this relationship further.

Fourth, as this study focused on the relationship between hyposensitivity and interoceptive processing in autistic adults, several measures differed significantly in previous studies were not examined. For example, we did not measure mood or arousal levels, which could affect interoceptive accuracy. The significantly higher heart rate in the ASD group than in the control group may reflect differences in mood and arousal levels. Furthermore, we did not examine about IA confidence, which was significantly different between autistic individuals and controls in previous studies [44].

Finally, while participants may have prior knowledge of their own heart rate as a reason for the lack of significant differences in interoceptive accuracy between the ASD and control groups, we did not collect data on participants’ prior knowledge of their own heart rate. Most previous studies have used regression analysis to control for individuals' prior knowledge of their heart rate (e.g., based on their smartwatches). Further investigations of these aspects are warranted.

### Supplementary Information

Below is the link to the electronic supplementary material.

**Supplementary file 1.** Correlation analysis of AQ with IA scores among the entire sample and ASD and control groups.

## Data Availability

Data sharing is not applicable to this article as no datasets were generated or analyzed in the current study. The corresponding author can be contacted for data requests.
