# Supplementary material for: The relationship between subjective difficulty in interoceptive processing and accuracy of heartbeat perception in autistic individuals
Source: Discov Ment Health. 2024 Apr 18;4(1):13. doi: 10.1007/s44192-024-00065-6 (PMC11026320; doi:10.1007/s44192-024-00065-6)
Supplement: Supplementary file 1 — Correlation analysis of AQ with IA scores among the entire sample and ASD and control groups. [file 44192_2024_65_MOESM1_ESM.docx]

**Supplemental Information**

Supplemental Results:

**Correlation analysis of AQ with IA scores among the entire sample and ASD and control groups.**

To investigate the relationship between AQ and IA scores, we computed Pearson's correlation coefficients. We found a significant correlation between total AQ and IA scores in the control group (r = 0.49, p = 0.03). However, there was no significant correlation between the IA scores and AQ in either the ASD group (r = 0.003, p = 0.99) or in the entire sample (r = 0.07, p = 0.37).

Supplementary Figure 1. Correlation between AQ and IA scores in ASD and Control groups.
